# Dietary Habits and Risk of Urothelial Cancer Incidence in the JACC Study

**DOI:** 10.2188/jea.15.S190

**Published:** 2005-08-18

**Authors:** Fumio Sakauchi, Mitsuru Mori, Masakazu Washio, Yoshiyuki Watanabe, Kotaro Ozasa, Kyohei Hayashi, Tsuneharu Miki, Masahiro Nakao, Kazuya Mikami, Yoshinori Ito, Kenji Wakai, Akiko Tamakoshi

**Affiliations:** 1 Department of Public Health, Sapporo Medical University School of Medicine.; 2 Department of Epidemiology for Community Health and Medicine, Kyoto Prefectural University of Medicine Graduate School of Medical Science.; 3 Department of Urology, Kyoto Prefectural University of Medicine Graduate School of Medical Science.; 4 Department of Public Health, Fujita Health University School of Health Sciences.; 5 Division of Epidemiology and Prevention, Aichi Cancer Center Research Institute.; 6 Department of Preventive Medicine/Biostatistics and Medical Decision Making, Nagoya University Graduate School of Medicine.

**Keywords:** Diet, urothelial neoplasms, Incidence, Cohort Studies

## Abstract

BACKGROUND: The relationships between dietary habits and urothelial cancer have been discussed in many epidemiologic studies, however, they have not been sufficiently elucidated. In the present study, the associations of dietary habits with the risk of urothelial cancer incidence were evaluated taking into consideration sex, age, and smoking habits.

METHODS: The Japan Collaborative Cohort Study (JACC Study) was planned in the late 1980s as a large-scale cohort study surveying people comprehensively and detailing their lifestyles, and the study subjects were followed up until the end of 1997. Among the total of 110,792 participants, 26,464 men and 38,720 women were in areas where incident cases with cancer were identified. During the observation period, 95 men and 28 women suffered from urothelial cancer. Hazard ratios for dietary factors were calculated by Cox’s proportional hazards model.

RESULTS: Increasing age, male gender, and smoking history were all significantly associated with the risk of urothelial cancer. High consumption of pork was significantly associated with the risk. In contrast, high intakes of milk and fresh fish were significantly inversely associated with the risk. High intakes of Chinese cabbage and fruits were also significantly inversely associated with the risk of urothelial cancer.

CONCLUSIONS: It is suggested that high intakes of milk, fresh fish, Chinese cabbage, and fruits have preventive effects against urothelial cancer.

Histologically the urothelium comprises the urinary bladder, ureter, and renal pelvis. Urothelial cancer generally originates in the mucosa of the lower urinary tract. The relationships between dietary habits and urothelial cancer have been discussed in many epidemiologic studies, but they have not been sufficiently elucidated. Recently, the protective activity of vegetables and fruits has been focused on with regard to antioxidative micronutrients such as carotenoids and vitamin C, and high consumption of vegetables and fruits is considered to reduce the risk of urothelial cancer.^1^^,^^2^ Additionally, it has been shown that experimental carcinogenesis in animals can be inhibited by many compounds from edible plants containing polyphenols, thiols, and glucocinolates.^3^

The Japan Collaborative Cohort Study (JACC Study) for Evaluation of Cancer Risk sponsored by the Ministry of Education, Science, Sports and Culture of Japan (Monbusho) was planned in the late 1980s as a large-scale cohort study surveying people comprehensively and detailing their lifestyles. Using data on dietary habits of this cohort study, we have already reported inverse associations of high intakes of milk and fruits with the risk of urothelial cancer death.^4^ In the present study, we analyzed the incidences of urothelial cancer to evaluate risk factors, because the mortality rate of urothelial cancer is in general rather low.

## METHODS

All data were taken from the JACC Study, the methods of which have been described in detail elsewhere.^5^ Briefly, the original study population consisted of 46,465 men and 64,327 women aged 40 to 79 years in 45 areas of 19 prefectures in Japan. Enrolment began in 1988 and continued until the end of 1990. Most subjects were recruited from the general population or when undergoing routine heath checks in the municipalities. The cohort was followed up until the end of 1997. All deceased persons in the study areas and persons who moved out of the study areas were identified using the population registry with the permission of each municipality office.

Incident cancer cases, 26,464 men and 38,720 women (58.8% of total participants in the JACC Study), were identified at 24 study areas in which cancer registries were available. The cancer registry system comprised reports from clinicians and death certificates; cancer cases with information from death certificate only (DCO) were included in incident cancer cases. The incidence including DCO from urothelial cancer was defined by codes C65, C66, and C67 in the ICD-10 (International Statistical Classification of Diseases and Related Health Problems, 10th Revision).

All participants completed a self-administered questionnaire included past and family histories, health conditions and lifestyle habits such as smoking, drinking, diet, physical exercise, occupation, and others. As for diet, the questionnaire elicited the intake frequencies of 32 food items. The subjects were asked about their average diet at the time of the baseline survey. The 32 items were pork, beef, chicken, ham and sausages, liver, eggs, fish (unprocessed), boiled fish paste (‘kamaboko’ in Japanese), dried or salted fish, milk, yogurt, cheese, butter, margarine, fried foods, fried vegetables, Chinese cabbage, cabbage and lettuce, green leafy vegetables, carrots and squash, tomatoes, edible wild plants (‘sansai’ in Japanese), mushrooms, potatoes, seaweed, pickles, foods boiled down in soy sauce (‘tsukudani’ in Japanese), boiled beans, tofu (soybean curd), oranges, fruits other than oranges, and fruit juice. There were five categories of frequency (seldom, 1-2 times a month, 1-2 times a week, 3-4 times a week, and almost every day). The results of the validity test for this questionnaire were briefly reported in a previous article.^6^

To ensure that the findings were not influenced by changes in dietary habits by participants with preclinical disease, we excluded all cases of urothelial cancer diagnosed during the first year of analysis. Accordingly, the subjects who suffered from urothelial cancer during the observation period were 95 males and 28 females (total 123 cases). Dead cases (29 cases) accounted for to 23.6% of total cases. Because female cases were comparatively few (22.8% of total cases), the hazards ratios (HRs) and 95% confidence intervals (CIs) for dietary factors were estimated among all subjects. The ratios were adjusted for age (numeric), and smoking index (number of cigarettes/day × number of smoking years). The effect was not adjusted for total energy because diet was measured by a simple food frequency method for limited food items. The five categories of food frequency were integrated into three groups by considering the number of subjects in each group and meaningful cutoff frequency. HRs of the highest and intermediate intakes compared with the lowest were calculated with Cox’s proportional hazards model using the PHREG procedure in the SAS^®^ (Statistical Analysis System) package. The HRs were obtained by stratification of observed regions using the ‘strata’ statement of the procedure, because diet may differ by area. The dose-response trend was tested by evaluating the regression coefficient when the three intake categories were treated as equally spaced numeric variables in Cox’s model. P values of less than 0.05 were considered significant. This investigation was approved by Ethical Boards of Nagoya University School of Medicine and Kyoto Prefectural University of Medicine.

## RESULTS

As primary localization, 12 patients had their tumor in the renal pelvis, 7 in the ureter, and 104 in the urinary bladder. The distribution of subjects for this study according to sex and age is shown in Table 1. The HR of men was about 4-fold that of women, and the risk of urothelial cancer increased for every 10-year increment in age (HR = 1.41, 95% CI: 1.28-1.55). Table 1 also presents HRs according to the smoking index after adjusting for sex and age. Subjects with 800+ cigarettes-years had a HR 2.75 (95% CI: 1.49-5.08) compared with nonsmokers. The HRs of urothelial cancer incidence dose dependently increased with the smoking index (p for trend = 0.002).

**Table 1.** Hazards ratios (HRs) of urothelial cancer and 95% confidence Intervals (CIs) according to sex, age, and smoking Index.

		Subjects (%)	Person-years	Cases	HR	95% CI
	Men	26,118 (40.5)	204,465	95	4.46	2.91-6.83
Sex	Women	38,421 (59.5)	296,388	28	1	(reference)
	Total	64,539 (100)		123		

	40-49	15,710 (23.5)	124,837	10		
Age	50-59	19,598 (30.4)	157,938	16		
	60-69	19,807 (30.7)	148,791	54		
	70-79	9,964 (15.4)	69,287	43		
	Total	64,539 (100)		Age(10-year increment)	1.41	1.28-1.55

	Non smokers	37,203 (64.5)	293,271	37	1	(reference)
Smoking*	Smoking index: 0-799	14,373 (24.9)	112,777	45	2.16	1.21-3.86
	Smoking index: 800+	6,096 (10.5)	46,132	34	2.75	1.49-5.08
	Total	57,672 (100)			trend P= 0.002	

*: Adjusted for sex and age.

Table 2 shows major findings on the relationships between consumption of various kinds of foods and HRs of urothelial cancer incidence after adjusting for sex, age, and smoking index. No significant associations of beef, chicken, ham and sausage, eggs, fish paste, or dried and salted fish consumption with the risk of urothelial cancer were found. However, intermediate consumption of pork was positively associated with the risk of urothelial cancer (HR = 1.85, 95% CI: 1.13-3.03), whereas intake of fresh fish almost every day was inversely associated with the risk (HR = 0.36, 95% CI: 0.18-0.72), with dose dependence (p for trend = 0.003). Regarding dairy products, high consumption of milk appeared to be inversely associated with the risk of urothelial cancer. The HR for the intermediate level of consumption of it compared with the lowest was 0.56 (95% CI: 0.33-0.97). However, consumption of other dairy products such as yogurt, cheese, and butter had no statistically significant association with the risk.

**Table 2.** Hazard ratios (HRs) of urothelial cancer for diet and 95% confidence intervals (CIs) adjusted for sex, age, and smoking index.

Item	Category	Person-years	Cases	HR	95% CI	Trend P
Pork	≦ 1-2/m	142,172	27	1	(reference)	
	1-2/w	208,684	64	1.85	1.13 - 3.03	
	3-4/w+	105,719	20	1.25	0.67 - 2.33	0.04

Beef	Seldom	111,147	24	1	(reference)	
	1-2/m	145,183	44	1.33	0.79 - 2.24	
	1-2/w+	160,663	34	1.17	0.66 - 2.05	0.64

Chicken	≦ 1-2/m	153,426	41	1	(reference)	
	1-2/w	207,090	59	1.32	0.86 - 2.02	
	3-4/w+	91,367	13	0.74	0.39 - 1.40	0.69

Ham & Sausages	≦ 1-2/m	215,328	47	1	(reference)	
	1-2/w	149,696	36	1.27	0.81 - 2.01	
	3-4/w+	66,013	19	1.40	0.81 - 2.43	0.18

Eggs	≦ 1-2/m	34,207	10	1	(reference)	
	1-2/w	104,833	31	0.83	0.40 - 1.73	
	3-4/w+	342,288	80	0.74	0.38 - 1.44	0.35

Fresh fish	≦ 1-2/m	42,277	16	1	(reference)	
	1-4/w	316,282	84	0.68	0.40 - 1.17	
	Almost every day	119,117	19	0.36	0.18 - 0.72	<0.01

Fish paste (*Kamaboko*)	≦ 1-2/m	223,874	59	1	(reference)	
	1-2/w	112,301	27	1.00	0.62 - 1.62	
	3-4/w+	47,641	9	0.79	0.38 - 1.63	0.60

Dried and salted fish	≦ 1-2/m	153,677	43	1	(reference)	
	1-2/w	176,437	41	0.88	0.55 - 1.39	
	3-4/w+	124,405	27	0.83	0.50 - 1.39	0.47

Milk	≦ 1-2/m	120,339	42	1	(reference)	
	1-4/w	125,194	21	0.56	0.33 - 0.97	
	Almost every day	219,390	52	0.65	0.42 - 1.01	0.06

Yogurt	≦ 1-2/m	297,083	72	1	(reference)	
	1-2/w	51,231	13	1.21	0.65 - 2.25	
	3-4/w+	44,256	8	0.82	0.39 - 1.71	0.79

Cheese	Seldom	195,792	53	1	(reference)	
	1-2/m	105,312	24	0.84	0.50 - 1.42	
	1-2/w+	85,909	22	0.90	0.54 - 1.52	0.63

Butter	Seldom	196,863	52	1	(reference)	
	1-2/m	92,980	23	1.14	0.68 - 1.90	
	1-2/w+	92,092	17	0.81	0.46 - 1.43	0.57

Chines cabbage	≦ 1-2/m	93,791	23	1	(reference)	
	1-4/w	249,181	70	0.85	0.52 - 1.38	
	Almost every day	72,993	14	0.48	0.24 - 0.98	0.05

Cabbage & lettuce	≦ 1-2/m	46,574	11	1	(reference)	
	1-2/w	145,436	45	1.39	0.71 - 2.70	
	3-4/w+	265,856	56	0.98	0.51 - 1.89	0.43

Green-leafy vegetables	≦ 1-2/m	42,259	14	1	(reference)	
	1-2/w	130,442	29	0.70	0.36 - 1.37	
	3-4/w+	271,703	65	0.74	0.41 - 1.36	0.52

Carrots & squash	≦ 1-2/m	75,556	23	1	(reference)	
	1-2/w	156,903	36	0.87	0.50 - 1.51	
	3-4/w+	227,743	58	1.01	0.60 - 1.71	0.83

Tomatoes	≦ 1-2/m	154,251	54	1	(reference)	
	1-2/w	137,650	23	0.62	0.37 - 1.02	
	3-4/w+	157,015	33	0.84	0.53 - 1.33	0.38

Oranges	≦ 1-2/w	172,008	58	1	(reference)	
	3-4/w	107,138	26	0.75	0.46 - 1.21	
	Almost every day	175,202	28	0.45	0.27 - 0.74	<0.01

Fruits other than oranges	≦ 1-2/w	156,987	53	1	(reference)	
	3-4/w	112,830	30	0.79	0.49 - 1.27	
	Almost everyday	175,334	25	0.45	0.27 - 0.76	<0.01

Abbreviations : 1-2 /m=once or twice a month, 1-2/w=once or twice a week, 1-4/w=once or 4 times a week, 3-4/w = 3-4 times a week.

With respect to vegetables and fruits, the highest intake of Chinese cabbage was inversely associated with the risk (HR = 0.48, 95% CI: 0.24-0.98), with dose dependence (p for trend = 0.046). Furthermore, high intakes of oranges and fruits other than oranges were also inversely associated with the risk (HR = 0.45, 95% CI: 0.27-0.74 for oranges, and HR = 0.45, 95% CI: 0.27-0.76 for fruits other than oranges), with dose dependence (p for trend = 0.02 for oranges, and 0.03 for fruits other than oranges). In the present study, intakes of green leafy vegetables, carrots and squash, and tomatoes appeared to be inversely associated with the risk of urothelial cancer incidence, however, their HRs were not statistically significant.

## DISCUSSION

It has been reported that bladder cancer occurs two- to fivefold more frequently in men than in women and its incidence increases with age.^7^ Our estimates of the HRs by sex and age were consistent with this previous report. Much epidemiologic evidence has shown that smoking increases the risk of urothelial cancer with a dose-response relation.^8^ In the present study the strength of the association between cigarette smoking and urothelial cancer was similar to the previous studies.

We also examined the associations of dietary habits with the risk of urothelial cancer incidence after adjusting for sex, age, and smoking index. Consumption of pork was positively associated with the risk of urothelial cancer. A high intake of pork has been reported to increase the risk of urothelial cancer, and it has been postulated that mutagens increasing the risk for bladder cancer are formed during cooking fat-rich meat like pork.^9^ Daily intake of fresh fish was significantly inversely associated with the risk. It was found that fish contains n-3 polyunsaturated fatty acids, which could reduce risk of several cancers.^10^ This may be one of possible explanations of the inverse association between high intake of fresh fish and the risk of urothelial cancer.

A high intake of milk was inversely associated with the risk of urothelial cancer incidence. This result was consistent with our previous finding that a high intake of milk was inversely associated with the risk of urothelial cancer death^4^. Thus, high intake of milk appeared to be preventive of the incidence and death from urothelial cancer. Milk is considered an important source of vitamin A, which has an antioxdative effect, and it contains lignans; Lignans are phytoestrogens that have been shown to possess anticarcinogenic activity. Recently it has been also reported that milk contains lactoferrin, which works as a chemopreventive agent.^11^

Some epidemiologic studies reported that frequent intake of fruits and vegetables was inversely associated with bladder cancer risk.^1^ Chinese cabbage belongs to the genus *Brassica*, including cruciferous vegetables such as broccoli. Cruciferous vegetables have been widely studied for their anticarcinogenic properties in experimental studies, and have been shown to be associated with reduced cancer risk. In fact consumption of cruciferous vegetables induces detoxification enzymes in animal tissues.^3^ Perhaps detoxification enzymes in Chinese cabbage may be important for prevention of urothelial cancer.

Unfortunately we could not observe a relationship between intake of green leafy vegetables, carrots, squash, or tomatoes and the urothelial cancer incidence. Some studies reported significant inverse associations between intake of dark green vegetables and the risk of bladder cancer.^1^ Carrots are rich in carotenoids, and carotenoids have been hypothesized to be anticarcinogenic agents. A well-designed study among Japanese will be needed to evaluate the effects of green leafy vegetables, carrots, squash, and tomatoes on urothelial cancer.

Intakes of oranges and fruits other than oranges were inversely associated with the risk of urothelial cancer incidence. These results also seemed to be consistent with our previous finding that a high intake of fruits was inversely associated with the risk of urothelial cancer death,^4^ and the idea that high consumption of vegetables and fruits is associated with decreased risk of cancer in most sites. The possible reason for the inverse associations is the protection afforded by antioxidants and other protective agents in fruits. In particular, citrus fruits like oranges contain vitamin C as an antioxidant; vitamin C plays an important role as a cancer-inhibiting agent.

In the present study we had the advantage of examining many incident cases with urothelial cancer (76.4% of total cases). Incidence rates provide the clearest measure to identify the exposure-outcome relationship at the population level^12^. Therefore it is important to analyze incident cases of cancer for which the mortality rate is in general rather low like urothelial cancer. Additionally we could find more preventive factors against urothelial cancer than in our previous study dealing with cases from death certificates.^4^ Daily intakes of fresh fish, Chinese cabbage, and oranges were considered to be preferable dietary habits in addition to intakes of milk and fruits other than oranges.

In conclusion, the present study was conducted to prospectively assess the effects of dietary habits on the risk of urothelial cancer. The most important factor was cigarette smoking as expected; thus, urothelial cancer could be potentially preventable by smoking cessation. Furthermore, it is suggested that high intakes of milk, fresh fish, Chinese cabbage, and fruits have preventive effects against urothelial cancer.

## MEMBER LIST OF THE JACC STUDY GROUP

The present investigators involved, with the co-authorship of this paper, in the JACC Study and their affiliations are as follows: Dr. Akiko Tamakoshi (present chairman of the study group), Nagoya University Graduate School of Medicine; Dr. Mitsuru Mori, Sapporo Medical University School of Medicine; Dr. Yutaka Motohashi, Akita University School of Medicine; Dr. Ichiro Tsuji, Tohoku University Graduate School of Medicine; Dr. Yosikazu Nakamura, Jichi Medical School; Dr. Hiroyasu Iso, Institute of Community Medicine, University of Tsukuba; Dr. Haruo Mikami, Chiba Cancer Center; Dr. Yutaka Inaba, Juntendo University School of Medicine; Dr. Yoshiharu Hoshiyama, University of Human Arts and Sciences; Dr. Hiroshi Suzuki, Niigata University School of Medicine; Dr. Hiroyuki Shimizu, Gifu University School of Medicine; Dr. Hideaki Toyoshima, Nagoya University Graduate School of Medicine; Dr. Kenji Wakai, Aichi Cancer Center Research Institute; Dr. Shinkan Tokudome, Nagoya City University Graduate School of Medical Sciences; Dr. Yoshinori Ito, Fujita Health University School of Health Sciences; Dr. Shuji Hashimoto, Fujita Health University School of Medicine; Dr. Shogo Kikuchi, Aichi Medical University School of Medicine; Dr. Akio Koizumi, Graduate School of Medicine and Faculty of Medicine, Kyoto University; Dr. Takashi Kawamura, Kyoto University Center for Student Health; Dr. Yoshiyuki Watanabe, Kyoto Prefectural University of Medicine Graduate School of Medical Science; Dr. Tsuneharu Miki, Graduate School of Medical Science, Kyoto Prefectural University of Medicine; Dr. Chigusa Date, Faculty of Human Environmental Sciences, Mukogawa Women’s University ; Dr. Kiyomi Sakata, Wakayama Medical University; Dr. Takayuki Nose, Tottori University Faculty of Medicine; Dr. Norihiko Hayakawa, Research Institute for Radiation Biology and Medicine, Hiroshima University; Dr. Takesumi Yoshimura, Fukuoka Institute of Health and Environmental Sciences; Dr. Akira Shibata, Kurume University School of Medicine; Dr. Naoyuki Okamoto, Kanagawa Cancer Center; Dr. Hideo Shio, Moriyama Municipal Hospital; Dr. Yoshiyuki Ohno, Asahi Rosai Hospital; Dr. Tomoyuki Kitagawa, Cancer Institute of the Japanese Foundation for Cancer Research; Dr. Toshio Kuroki, Gifu University; and Dr. Kazuo Tajima, Aichi Cancer Center Research Institute.
